# Supplementary material for: Patterns of Intron Gain and Loss in Fungi
Source: PLoS Biol. 2004 Nov 30;2(12):e422. doi: 10.1371/journal.pbio.0020422 (PMC532390; doi:10.1371/journal.pbio.0020422)
Supplement: Table S1 — Also available at http://genes.mit.edu/NielsenEtAl/. (4.3 MB ZIP). [file pbio.0020422.st001.zip › NielsenEtAl/html/692.html]

AN5705.1.NCU06091.1.MG00839.1.FG06346.1


```
 CLUSTAL W (1.82) Multiple Sequence Alignments - Introns Inserted


Sequence 1: MG00839.1	366 aa
Sequence 2: FG06346.1	349 aa
Sequence 3: NCU06091.1	349 aa
Sequence 4: AN5705.1	353 aa
Alignment Length: 369 aa
Number Identitical Residues: 241 aa
Alignment Score (without introns) 10089


MG00839.1 	MSTNASGGLTRRRGGGGGGGGGGAGAGGDEGSSSNAGAIGRSDSSSNFQKESSSSAPETS
NCU06091.1	MAS--TSGLTRRRGAGGG--------------SSDVGGGGGGGGAS----DTRDVGPDTS
FG06346.1 	MSS----GLTRRRGAGGAGPAG-------EGDSSN-GAAPRTSTPS-----ARDNGPETS
AN5705.1  	MAS--AGGLTRRRGGGRATG------------TDDHDDSRVSSPVSRNGSAMDNRIPETS
          	*::  :.*******.* .              :.: .     .  *   .   .  *:**

MG00839.1 	YEKGENGHKIAFDPRDISESAERSKQPKLTLMEEVLLLGLKDKQ~GYLSFWNDNISYALR
NCU06091.1	YETGENGHRIAFDPRDLSESAERSKQPKLTLMEEVLLLGIKDRE0GYLSFWNDNISYALR
FG06346.1 	YENSENGHKIAFDPRDISESAERSKQPKLTLMEEVLLLGLKDKQ0GYLSFWNDNISYALR
AN5705.1  	YTNSENGHKIAFDPRDICETQERSKQPKLTLMEEVLLLGLKDKQ0GYLSFWNENISYALR
          	* ..****:*******:.*: ******************:**:: *******:*******

MG00839.1 	GCIVIELAFRGRISMQKDPSRRRFPLADRLVEVIDDSLTGEVLLDEALKLMKSSEKMSVS
NCU06091.1	GCIVLELAFRGRISMQKDSSRRRFPLADRIIEVIDDTLTGEVLLDEALKMMKTSEKMSVS
FG06346.1 	GCIVIELAFRGRIAMEKDASRRRFPLADRNIEVIDDTLTGEVLLDEALKMMKQSEKMSVA
AN5705.1  	GCIVIELALRGRVSMQKDSSRRRFPLADRVIEVIDDTLTGEVLLDEALKMMKSSEKMSVN
          	****:***:***::*:**.********** :*****:************:** ****** 

MG00839.1 	SWIDLLSG1ETWNLMKIGYQLKQVRERLAKGLVDKGILRTEKRNFLLFDMATHPVVDGGA
NCU06091.1	SWIDLMSG1ETWNLMKIGYQLKQVRERLAKGLVDKGILRTEKRNFLLFDMATHPVVEGAA
FG06346.1 	TWIDLMSG1ETWNLMKIGYQLKQVRERLAKGLVDKGILRTEKRNFLLFDMATHPVADGGA
AN5705.1  	SWIDLMSG1ETWNLMKIGYQLKQVRERLAKGLVDKGILRTEKRNFLLFDMATHPVADGGA
          	:****:** **********************************************.:*.*

MG00839.1 	KEEIRRRVRNVLTQRTVVLPGSQFLPENLEFRYSRTIALVCAAYAANVLENALSTLGHEA
NCU06091.1	KEDIRRRVRNVLTQRTVVLTPTQYLPEDLEFRYVRTIAMVCAAHAANVLENALSTLGHEA
FG06346.1 	KEEIRRRVRTVLTQRTVVLNGSQFLPESLEFRYMRTIAMVCAAYAANVLENALASLGHEA
AN5705.1  	KEDLNRRVRSICSSRTVILPANQWLPEDIEFRYLRTITMVCAAYAANVLENALVTMSHEA
          	**::.****.: :.***:*  .*:***.:**** ***::****:********* ::.***

MG00839.1 	RERAFAQTDELLAEYSQWPFGKKATAHG---IGANLPQVISD0EIAKGKDKELQLE~VVA
NCU06091.1	RENAFSMADELLLQYSQWPFAARPLPGGNGGVGANLPQVINE0EVNKAKDKELQLE~VVA
FG06346.1 	RERAFAQTDDLLADYSQWPFGKKATQNG---VGANLPQLINE0EVNSGKDKELQLE~VVS
AN5705.1  	RERAFAQVDELLAEYSQWPFARRPGGSQS--IGANLAQAIND~EVNKNSDKELQLE0VVA
          	**.**: .*:** :******. :.    .  :****.* *.: *: . .******* **:

MG00839.1 	ACLSVFTRLDSLL
NCU06091.1	ACLSVFTRLDSLL
FG06346.1 	ACLSVFTRLDSLL
AN5705.1  	ACLSVFTRLDSLL
          	*************
```
